# Prevalence of Canine Hip Dysplasia in Switzerland Between 1995 and 2016—A Retrospective Study in 5 Common Large Breeds

**DOI:** 10.3389/fvets.2019.00378

**Published:** 2019-10-24

**Authors:** Stefanie Ohlerth, Britta Geiser, Mark Flückiger, Urs Geissbühler

**Affiliations:** ^1^Clinic for Diagnostic Imaging, Department of Clinical Services and Diagnostics, University of Zurich, Zurich, Switzerland; ^2^Section of Clinical Radiology, Department of Clinical Veterinary Medicine, University of Bern, Bern, Switzerland

**Keywords:** Bernese mountain dog, dog, canine hip dysplasia, German shepherd dog, prevalence, retriever

## Abstract

Canine hip dysplasia (CHD) is a genetic disease, modulated by epigenetic and environmental factors. To decrease the prevalence of CHD, the hip joints of many pure breed dogs are radiographed to identify dysplastic dogs not qualified for breeding. It has been shown that both, prevalence and severity of CHD may be reduced on the basis of phenotypic i.e., radiographic selection of breeding animals. The method has been used in many countries for over 50 years. In the present study, severity and prevalence of CHD in five common large breeds in Switzerland were evaluated since 1995. Both, prevalence and severity of CHD dropped in each breed between the periods 1995–1999 and 2010–2016. The prevalence decreased in Golden Retrievers from 25 to 9% and in Labrador Retrievers from 16 to 3%, respectively. In the Flat-Coated Retriever, prevalence in general was low, decreasing from 6 to 3%. In the Bernese mountain dog and the German shepherd dog, a decrease from 21 to 12% and from 46 to 18%, respectively, was observed. However, the rather low overall rate of radiographed dogs (Retrievers: 11 to 18%, Bernese Mountain dogs: 23% and German Shepherd dogs: 31%) does not allow to draw reliable conclusions regarding the true prevalence of CHD for the entire population of these breeds in Switzerland.

## Introduction

Canine hip dysplasia (CHD) is defined as a developmental disease of the coxofemoral joint (1) and occurs in most canine breeds. The joint appears normal and congruent at birth but develops abnormal during growth. Excessive laxity is assumed to be the key factor leading to subluxation of the femoral head, incongruity of the joint and subsequent flattening of the acetabulum (2). As a result, the load on the cartilage is unevenly distributed resulting in unequal cartilage abrasion, followed by an inflammatory response and finally secondary degenerative joint disease, causing pain and lameness (1, 3). Presence and degree of CHD may be assessed on the basis of radiographic changes, i.e., subluxation, joint deformation, and osteoarthritis (4).

Canine hip dysplasia is a multifactorial disease triggered by genetic, environmental and probably epigenetic factors (5, 6). The genetic basis of CHD is not fully understood; however, it is assumed to be a complex genetic trait with a polygenic inheritance pattern. Both, dominant and recessive modes of inheritance have been discussed (7, 8). Due to its genetic background, excluding affected dogs from breeding may reduce the prevalence of CHD. The heritability of CHD and therefore the response to selection is breed dependent. The higher the heritability of a trait, the greater is the expected genetic improvement over time when selective breeding is practiced (9, 10). Breeding stock is usually selected based on the radiographic phenotype.

The radiographic projection in dorsal recumbency with extended hip joints is used for assessment of CHD in most countries (11). In Switzerland, a second view with flexed and abducted stifles is mandatory for official scoring to improve scoring quality (12). Minimum age of the dogs for official scoring is between 12 and 24 months, depending on the country and the scoring method used.

Prevalence of CHD has been reported to vary significantly between breeds and countries. In France, the prevalence varied between 3.9% (Siberian Husky) and 59.7% (Cane Corso) over the period 1993–2006 (13). In the United States, values of 1.5% in the Miniature Schnauzer and 35.4% in Rottweilers were reported between 1991 and 1995 (14). In Switzerland between 1991 and 1994, the CHD-prevalence ranged from 7% in Siberian Huskies to 69% in Gordon Setters. For popular breeds such as the Retrievers, the Bernese mountain dog and the German shepherd dog, prevalence of CHD was in the range of 31–53% (15). During the last decades many selective breeding programs based on the radiographic scoring have been implemented for different breeds with the aim to reduce the prevalence and severity of CHD, and consequently, to improve animal's welfare. A decrease of the CHD-prevalence was noted in some reports (13, 16, 17); however, progress was slow or inexistent in others (18, 19).

Reports of the recent prevalence of CHD in Switzerland are lacking in the peer-reviewed literature. The aim of the present study was therefore to assess the overall prevalence of CHD and its change since 1995 in five common large dog breeds in Switzerland.

## Materials and Methods

### Animals

The largest purebred dog populations in Switzerland i.e., Golden Retrievers, Labrador Retrievers, Flat Coated Retrievers, Bernese Mountain dogs and German Shepherd dogs were included in the study. The Swiss kennel clubs provided the official CHD score and the date of birth for each dog. The vast majority of the dogs were examined in their second year of life.

### Scoring Protocols

Most dogs were scored according to the Swiss scoring system (4). Ninety-six imported dogs had been scored abroad; for the present study their scores were transferred to the Swiss system as shown in Table 1.

**Table 1:** Comparison of scoring protocols for canine hip dysplasia.

**Switzerland (point score per joint)**	**FCI**	**BVA/KC (point score per joint)**	**OFA**
0–2	A: normal	0–3	Excellent
3–6	B: near normal	4–8	Good
7–12	C: mild CHD	9–18	Fair, borderline, mild
13–18	D: moderate CHD	>18	Moderate
>18	E: severe CHD		Severe

*FCI, Fédération Cynologique Internationale, BVA/KC, British Veterinary Association/The Kennel Club, OFA, Orthopedic Foundation for Animals*.

The Orthopedic Foundation for Animals (OFA) system is used in the USA and Canada. Minimum age for scoring is 2 years. Seven grades are defined: excellent, good, fair, borderline, mild, moderate, or severe. The borderline grade is assigned to incongruent joints of undetermined quality but without degenerative changes. The British Veterinary Association/The Kennel Club (BVA/KC) system is used in Britain, Ireland, Australia and New Zealand. Dogs older than 12 months are evaluated. Nine radiographic criteria are evaluated; each rated with 0–5, or 0–6 points, respectively (20, 21). A total between 0 and 52 points is allotted to each joint. In Britain, the points per joint are added up representing the final score whereas in Australia only the total points of the worse hip joint is used for the final score (22). The Fédération Cynologique Internationale (FCI) system is used in most European countries, Russia, South America, and Asia (23). Minimum age for official scoring is 12 months, in giant breeds 18 months (24). Each joint is allotted to one of five grades (A–E) that are defined descriptively; the final grade refers to the worse joint. In Switzerland a system is used linking the British system with the FCI grading system allowing a more systematic and objective scoring. The same six radiographic criteria as in the FCI system are evaluated; each criterion is allotted 0–5 points leading to a sum of 0–30 points per joint. Criteria 1 and 2 quantify the degree of laxity. Criteria 3 and 4 determine modeling of the acetabulum and criteria 5 and 6 include arthritic changes of the femoral head and neck (4). The numeric score is then translated into the FCI-grades A–E (23). For the Bernese mountain dog the minimal age for official scoring in Switzerland is 14 months, whereas it is 12 months for the Retriever breeds and the German shepherd dog.

### Statistical Analysis

The kennel clubs provided data on birth rate and official CHD score. Descriptive statistics was performed using the SPSS statistics program (Version 19, IBM Corporation, Armonk, New York). Hip dysplasia grades A and B were considered normal joints (CHD-free) whereas the grades C, D, and E were considered dysplastic.

## Results

**Figure 1:**
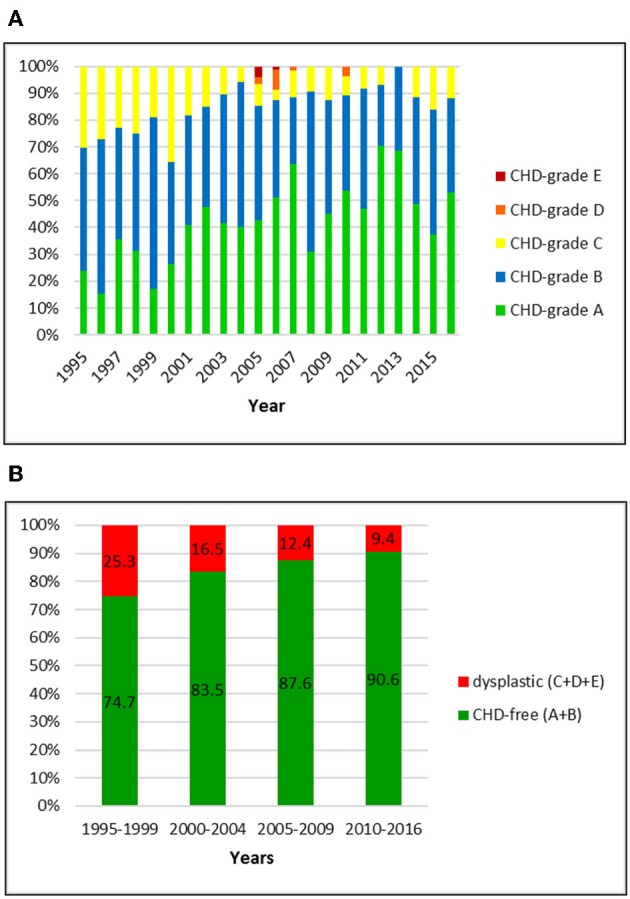
**(A)** Distribution of CHD-grades in the Swiss Golden Retriever population from 1995 to 2016. **(B)** Proportion of dysplastic and CHD-free Golden Retrievers in Switzerland from 1995 to 2016: the prevalence of CHD dropped markedly over 22 years.

**Figure 2:**
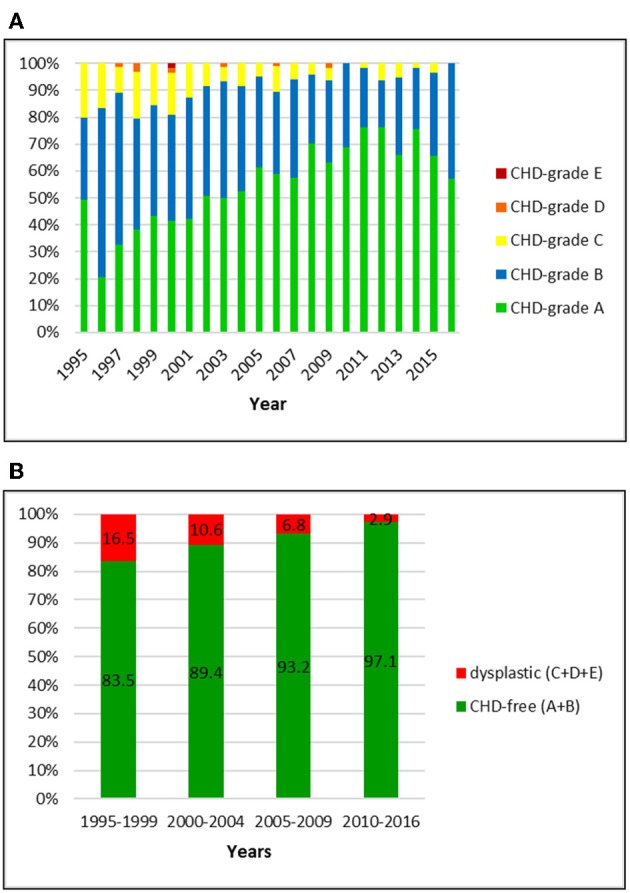
**(A)** Distribution of CHD-grades in the Swiss Labrador Retriever population from 1995 to 2016. **(B)** Proportion of dysplastic and CHD-free Labrador Retrievers in Switzerland from 1995 to 2016: the prevalence of CHD decreased over 22 years.

**Figure 3:**
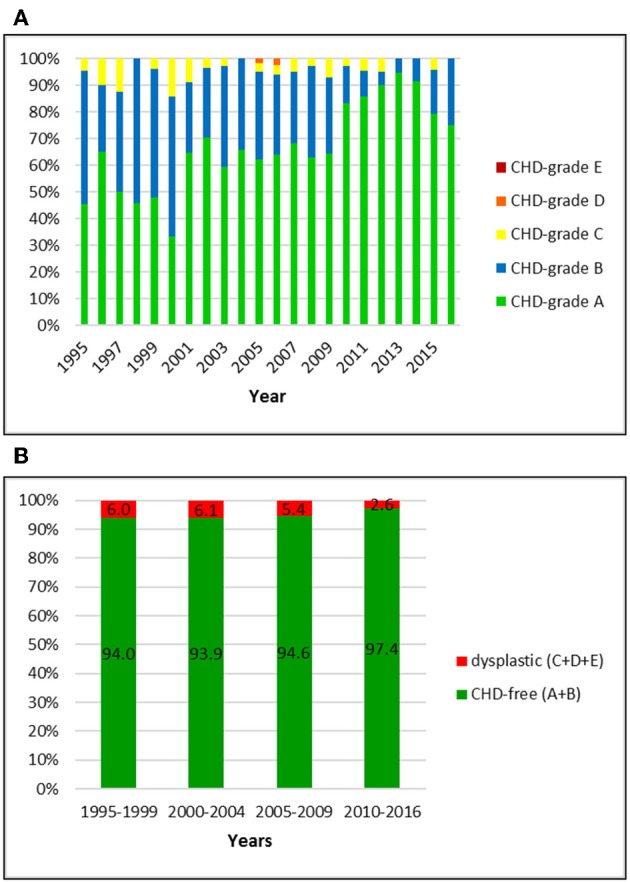
**(A)** Distribution of CHD-grades in the Swiss Flat-Coated Retriever population from 1995 to 2016. **(B)** Proportion of dysplastic and CHD-free Flat-Coated Retrievers in Switzerland from 1995 to 2016: in comparison to the other breeds, the prevalence of CHD has been markedly lower and values stayed constant during the study period.

**Figure 4:**
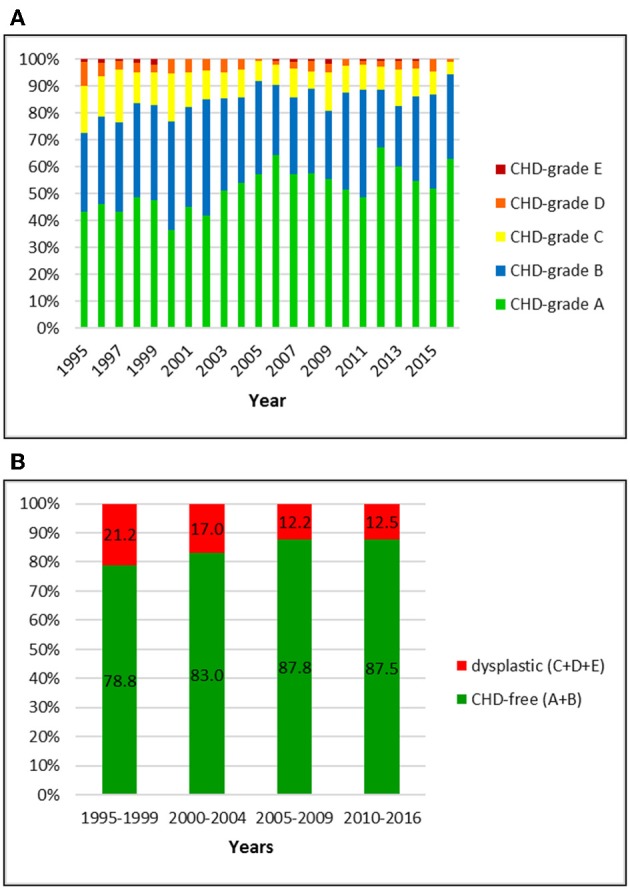
(**A)** Distribution of CHD-grades in the Bernese mountain dog population in Switzerland from 1995 to 2016. **(B)** Proportion of dysplastic and CHD-free Bernese mountain dogs in Switzerland from 1995 to 2016: the prevalence of CHD mildly decreased over 22 years.

**Figure 5:**
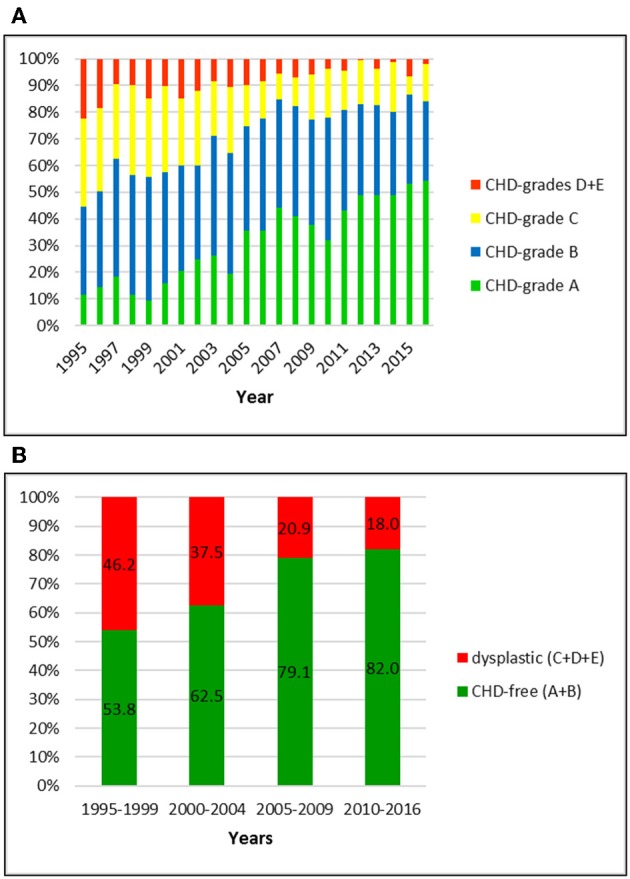
**(A)** Distribution of CHD-grades in the German shepherd dog population in Switzerland from 1995 to 2016. **(B)** Proportion of dysplastic and CHD-free German shepherd dogs in Switzerland from 1995 to 2016: the prevalence of CHD was initially the highest and showed the largest decline over 22 years compared to the other investigated breeds.

Prevalence of the five CHD-grades and prevalence of dysplastic dogs are shown for each breed from 1995–2016 in Figures 1–5.

### Golden Retriever

The Swiss retriever club registered an official CHD-score for 1047 Golden Retrievers between 1995 and 2016. Of these dogs, 27 (2.6%) were scored abroad (BVA/KC system). The overall prevalence of CHD was 15.8% including 14.4, 1.0, and 0.4% dogs with grade C, D, and E, respectively. Of the 84.2% CHD-free dogs, 41.6% were scored grade A and 42.6% grade B, respectively. In the first period (1995–1999), 25.3% of the Golden Retrievers were dysplastic whereas in the final period (2010–2016) the prevalence of CHD had dropped to 9.4%. Most remarkable were the decrease of dogs with grade C (from 25.3 to 9.0%) and the increase of A-scored dogs (from 24.1 to 53.5%). The fraction of A and B joints reached ~90% in 2004 and remained unchanged ever since.

### Labrador Retriever

Official data of 1512 Labrador Retrievers were available for the period from 1995 to 2016. Of these, 51 (3.4%) had a BVA/ KC-score and six (0.4%) dogs an OFA-score that was translated into the Swiss system. The overall prevalence of CHD was 9.1%, including 8.5, 0.5, and 0.1% of dogs with grade C, D, and E, respectively. An A-score was allotted to 54.6% and grade B to 36.3%. In the initial period, 16.5% were scored dysplastic. The number dropped to 2.9% in the latest period 2010–2016. There was a remarkable decrease of C-rated dogs (15.7–2.9%) and an increase of A-rated dogs (36.8–70.6%). More than 90% of the dogs were CHD-free since 2006.

### Flat-Coated Retriever

Overall, 768 Flat-Coated Retriever with an official CHD-score were registered by the Swiss Retriever Club between 1995 and 2016, of which three (0.4%) were BVA-rated and then translated into the Swiss system. The overall CHD-prevalence of 5.0% included 4.6% with grades C, 0.4% with grade D and 0% with grade E, respectively. The proportion of CHD-free dogs was 95.0%; of these, 65.7% were rated grade A and 29.3% grade B, respectively. The proportion of dysplastic dogs was initially 6.0% and dropped to 2.6% in the period 2010–2016. Simultaneously, the proportion of C-rated dogs dropped from 6.0 to 2.6% and the percentage of A-rated dogs increased from 50.0 to 86.6% in these years. Responsible for this noticeable increase of A-rated dogs was not only a reduction of C-dogs but also a decrease of B-rated dogs: from 44.0% in 1995–1999 to 10.8% in the final period.

### Bernese Mountain Dog

The Swiss Club for Bernese Mountain Dogs registered 3,381 dogs with an official CHD-score between 1995 and 2016. Nine (0.3%) dogs were BVA/KC-rated.

Dysplastic hip joints were noted in 15.7% of the dogs, including 11.6, 3.5, and 0.6% of dogs with grade C, D, and E, respectively. Of the CHD-free dogs (84.3%), 51.6% were rated as CHD-A and 32.7% as CHD-B. In the initial period (1995–1999) the CHD-prevalence was 21.2%. It dropped to 12.5% in 2010–2016. While C-rated hip joints decreased from 15.2 to 9.5%, A-rated dogs increased from 45.8 to 56.7% in these years.

### German Shepherd Dog

Data of 5326 German shepherd dogs was available for the study period. All dogs were scored by the Swiss system. The overall prevalence of CHD was 32.4%; of these, 22.5% were scored grade C and 9.9% grade D or E, respectively. Grade D and E were summarized in the database of the breeding club and therefore cannot be shown separately. The proportion of CHD-free dogs was 67.6%, including 27.9 and 39.7% of dogs with grade A and B, respectively. When compared to the other breeds, German shepherd dogs showed the highest prevalence of CHD initially followed by the steepest decline over 22 years. While 46.2% of them were dysplastic between 1995 and 1999, the number dropped to 18.0% in the period 2010–2016. In particular, the number of A-rated dogs increased from 13.0 to 46.5%, and the number of C-rated dogs decreased from 31.1 to 14.8%.

### Scoring Rate

**Figure 6:**
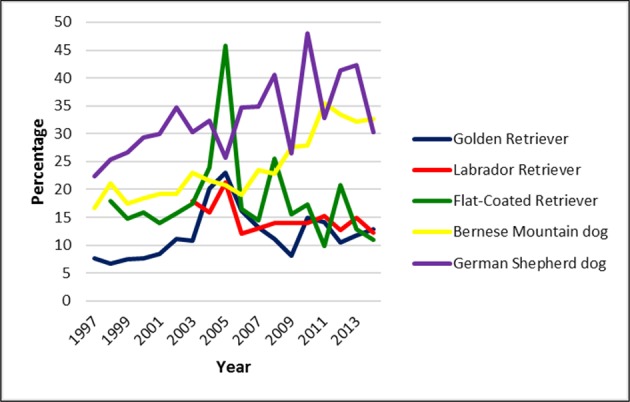
Scoring rate in five common large breeds in Switzerland from 1995 to 2016. It was lowest in the Golden Retriever and highest in the German shepherd dog. However, overall scoring rate was low.

The rate of radiographed dogs was calculated based on the number of puppies born per year. Offspring statistics of the examined breeds were not available for the entire study period. In the Golden Retriever, 7,947 puppies were registered between 1997 and 2015. Of these 881 dogs were screened for CHD, representing a scoring rate of 11.1%. In the Labrador retriever, litter information was available for the period between 2003 and 2016. Of 6,155 dogs born, 866 (14.1%) were officially scored for CHD. The scoring rate for the 3819 Flat-Coated Retrievers registered between 1998 and 2015 was 17.3% (659 dogs). In the Bernese mountain dog, 12,565 puppies were born between 1997 and 2015 of which 2,863 (22.8%) underwent official CHD screening. The highest scoring rate was noted in the German shepherd dogs: 13,998 dogs were born between 1997 and 2015 and 4,327 dogs were officially scored, equalling a scoring rate of 30.9%. Scoring rate per year is shown for each breed in Figure 6.

## Discussion

Control of CHD in Switzerland started around 1965. Obviously dysplastic dogs i.e., score D or E have been banned from breeding in Switzerland since more than 50 years. Nevertheless, the CHD-rate remained very high for a long time reaching levels above 50% in some breeds. Between 1991 and 1994, the CHD-prevalence was 51% in Golden Retrievers, 42% in Labrador Retrievers, 31% in Flat-Coated Retrievers, 46% in Bernese mountain dogs and 53% in German shepherd dogs, respectively (15). Since then, between 1995 and 2016, the CHD-prevalence has dropped considerably in all five breeds. In particular, there was a remarkable decrease of C-graded dogs and, inversely, an increase of A-graded dogs in all breeds. Whereas, grades D and E occurred mainly in the Bernese mountain dog and the German shepherd dog during the first decade of the investigated period of the present study, these scores were very rare in the Retriever breeds. During the second decade, it is interesting to note that in the German shepherd dog a continuous mild improvement of the hip joint quality was observed whereas the CHD-prevalence was already on a constant low level in the other investigated breeds. In other countries a similar but less pronounced decrease of CHD-prevalence has been reported. In France a significant decrease of CHD in six of 15 breeds was noted, e.g., the overall prevalence dropped from 27% in 1993–1999 to 19% in 2000–2006 in the Bernese mountain dog (13). In comparison, the overall CHD-prevalence in the Swiss population of Bernese mountain dogs dropped from 21% in 1995–1999 to 12.5% in 2010–2016. CHD-prevalence in Switzerland started on a lower level and the reduction was more pronounced, but observation time was also longer compared to the French study.

In the United States, a study between 1993 and 2003 showed only mild to no improvement. The rate of dysplastic Bernese mountain dogs dropped from about 16–12%, that of Labrador Retrievers from 12 to 9.5% and that of the Golden Retrievers from 18 to 15.5%, while the rate for German shepherd dogs oscillated between 11 and 19% with no clear improvement. Scoring rate was 5–7% for Retrievers and German shepherd dogs while it was roughly 24–34% for Bernese mountain dogs (25). In Finland no improvement was noted in Golden Retrievers, Labrador Retrievers and German shepherd dogs between 1983 and 1998 (19). In South African Labrador Retrievers only a minor improvement was seen between 2007 and 2015 (26). It was beyond the scope of the present study to investigate variables leading to a decrease in CHD-prevalence in the investigated breeds; however, several hypotheses may be discussed. Cross-national general factors may be responsible such as increasing awareness for inherited diseases by the kennel clubs, breeders and dog buyers on the one hand and improved training i.e., higher qualification of the scrutineers on the other hand. Vice versa, the lack of breeding restrictions associated with a lower scoring rate may be, in part, responsible for a lower progress in other countries when compared to the results of the present study. In Switzerland, for example, pairing C-graded dogs has been banned 10 years ago in German shepherd dogs, and in the Bernese mountain dog club, C-dogs may only be paired with A-dogs (https://www.retriever.ch/de/zucht/ankoerung; accessed July 21, 2019; https://www.bernersennenhund.ch/chronik; accessed July 21, 2019). Additionally, the Swiss registry may have helped to improve genetic hip quality because breeding dogs with better hips have been selected from the registered pool.

Furthermore, the scoring system in Switzerland is different from other countries. In all breeds, hip joint radiographs for official scoring are submitted to and evaluated by two independent committees only. Additionally, the use of the point scoring system by Flückiger introduced in the early nineties (4) may have led to a more objective and stricter evaluation process. Differences in the prevalence and the course were also noted between breeds in the present study. Varying heritability, rate of imported breeding dogs with different genome, and overall breeding regimen e.g., selection for other genetic diseases may have played a role.

Currently a purely phenotypic selection mode against hip dysplasia is used in most breeding clubs worldwide. As long as no breeding restrictions are enforced it is very unlikely that the dysplasia rate of offspring can be lowered much further based on this modality alone. Further improvement may be expected by several approaches. The key methods are hip joint laxity measurement, calculation of estimated breeding values, rigid offspring control and genomic selection.

Hip joint laxity is considered the key factor for the development of CHD. The standard hip extended ventrodorsal projection masks hip joint laxity and the degree of laxity may be quantified by radiographic techniques such as the PennHIP method, the Fluckiger method or the dorsolateral subluxation test (27, 28, 28–30). Heritability estimates of the hip-extended score as well as hip joint laxity measurements have been shown to be high (31). However, according to a recent study, the Norberg angle was not an accurate predictor of canine hip conformation based on the distraction index and the dorsolateral subluxation score. Authors suggested that application of screening methods for CHD based on hip laxity (intermediate phenotype screening) would help to remove additional dysplastic dogs from the breeding pool (32).

However, the method is also confronted with multiple obstacles. PennHIP requires a special training and tool and submission of the radiographs to the company holding the copyright. Strict adherence to the proposed selection suggestions (27) would also result in gene loss as more than half of the tested dogs fail the test in some breeds. Breeders must control additional undesirable diseases as well such as elbow dysplasia, eye diseases, epilepsy, cancer, skin disorders and others. Breeders are also concerned about possible damage to the hip joints and therefore refuse implementation of a distraction technique. However, there is no data indicating that application of a distraction method affects the natural evolution of hip dysplasia and one method, the dorsolateral subluxation test (28), has been shown not to place additional stress on the canine hip above walking and running load.

Breed value estimation (BVE) has been proven to be highly successful in livestock such as cattle, swine, and poultry. Several studies estimated that the introduction of BVE in dogs imposes a more severe downward pressure on hip dysplasia prevalence than eye balling a pedigree (18, 20, 33–36). The estimated breeding value is calculated from the phenotype of an individual and its relatives and their pedigree relationship (21). Including information about the hip status of relatives has been proven to be an efficient selection mode in mass selection, as the mode of inheritance of CHD is still unclear and dogs with phenotypic normal hip joints may carry undesirable genes passing CHD to their offspring (21). The effect of BVE however is weak or even counterproductive when affected dogs are sorted out prior to official scoring, which is not uncommon practice in German shepherd dogs worldwide. To profit from BVE, a rigid unbiased offspring control should be installed. Breeding animals with poor quality offspring should be banned irrespective of their own hip status (37). Currently, offspring control rates are low, as radiography is quite expensive and deep sedation or general anesthesia is mandatory for the official radiological procedure. Owners are generally reluctant to have their dogs anesthetised as long as they are not intended for breeding and show no clinical signs.

Offspring control is an efficient way to reduce CHD. The Swiss School for Guide dogs for the Blind achieved impressive results after including offspring testing in their mass selection. The CHD-prevalence of their Labrador Retrievers dropped from 58% in the years 1972–1980 to 15% in the period 1991–1996 (38). The current dysplasia rate is <3%, and of these no D or E grades are noted (unpublished data, Dysplasia Committee Zurich 2016 and 2017). The school is scoring virtually 100% of the dogs and adheres to a strict selection scheme. Only dogs with CHD-grades A and B are used for breeding and information of the relatives are used when selecting potential breeding animals. Phenotypically normal sires are eliminated from the breeding stock if their offspring turns out to be dysplastic. Providing free access of offspring data to the public is a supplementary way to improve hip quality. The Swiss Bernese Mountain dog club has been publishing the offspring grades of each breeding sire since 1990, as soon as at least 10 offspring have been controlled (https://www.bernersennenhund.ch/chronik; accessed Sept 9, 2019). Offering this information allows breeders to exclude sires producing an excessive number of affected offspring from the breeding stock. Collection and publication of all data available, including those of lame elder dogs, should also be encouraged.

As CHD is a hereditary disease, several attempts have been made to localize the responsible genes (39). A genomic analysis should increase the accuracy of BVE and thus reduce the rate of CHD (40, 41). Compared to phenotypic selection, genomic selection has the advantage that the blood test is relatively cheap and can be done immediately after birth (42). This would allow breeders to keep valuable dogs in stock for later breeding. Currently genomic selection is not possible since the chromosome location of the genes determining hip conformation remains largely unknown (43). Hip conformation seems to be based on many genes with small effects, so that marker-assisted selection may not be successful either (44). A commercially available test for German shepherd dogs failed to show any positive effects and was considered unsuitable for CHD risk assessment (43). More research in different breeds is needed to establish genetic tests for early diagnosis and mass screening of puppies (42).

The overall scoring rate oscillated somewhat over the years. In Golden and Labrador retriever the rate was low (<15%) and dropped further. In Bernese Mountain dogs it was almost twice as high and increased slightly over the past few years. The Swiss Bernese Mountain Dog club established a health fund in 1999 to encourage its members to actively control the health status of their dogs (http://www.bernersennenhund.ch/club). This may have helped to increase the scoring rate. The highest scoring rate was noted in the German shepherd dog. This may be associated with the common use of German Shepherds as working or sport dogs. However, whereas in German speaking countries i.e., Austria, Germany and Switzerland, all Retriever, Bernese Mountain and German shepherd dogs considered for breeding must be screened for CHD, in most other countries hip control is not mandatory and therefore only a small fraction of all breeding dogs is radiographed (26). In the UK for example selection of dogs used for breeding is left to the discretion of the owners with no restriction whatsoever (https://www.bva.co.uk/canine-health-schemes/hip-scheme/, accessed Sept 9, 2019). In the US no mandatory hip scoring is installed and the OFA registry is based on voluntary reporting (45, 46).

Some limitations of our results should be addressed. The scrutinizers in the two dysplasia committees varied over the entire study period although more than three quarters of all dogs were scored by the same four experts. Personal experience and new knowledge acquired over the years may also have influenced the scoring mode (47). The key limitation in most studies including the present one is the selection bias of the raw data (37, 45, 46, 48). Radiographs of dogs with clinical or obvious radiographic signs of CHD are less commonly submitted for official evaluation (45). This leads to an underrating of the true CHD-prevalence in the population. The true CHD-prevalence can therefore not be determined by scoring potential breeding stock only. As a consequence the Swiss Bernese Mountain Club changed their regulation in 2011 and decided that from every litter a particular number of random chosen dogs has to be evaluated for CHD (https://www.bernersennenhund.ch/reglemente-statuten, accessed Sept 9, 2019). Lack of data on dogs that were treated or euthanized because of CHD during the first year of life also is a strong bias. Lastly, radiographic evidence of hip osteoarthritis takes time to develop, and screening at 1 year of age based on the hip-extended view inevitably misses later onset and subtle radiographic signs of CHD.

In conclusion, the present study confirms that the prevalence of CHD could be reduced efficiently in five common large breeds in Switzerland over the last two decades using a systematic and strict phenotypic scoring scheme. However, the true prevalence of CHD is probably higher than reported. To put more downward pressure on the incidence and prevalence of CHD, additional programs should be considered such as breeding only dogs that are screened by a veterinarian and are publicly available, intermediate phenotype screening (measurement of joint laxity) and EBVs.

## Data Availability Statement

The datasets generated for this study are available on request to the corresponding author.

## Author Contributions

All the authors contributed to the conception or design of the work, drafting and revising, and final approval of the version to be published.

### Conflict of Interest

The authors declare that the research was conducted in the absence of any commercial or financial relationships that could be construed as a potential conflict of interest.
